# Gs-DREADD Knock-In Mice for Tissue-Specific, Temporal Stimulation of Cyclic AMP Signaling

**DOI:** 10.1128/MCB.00584-16

**Published:** 2017-04-14

**Authors:** Dmitry Akhmedov, Maria G. Mendoza-Rodriguez, Kavitha Rajendran, Mario Rossi, Jürgen Wess, Rebecca Berdeaux

**Affiliations:** a Department of Integrative Biology and Pharmacology, McGovern Medical School at The University of Texas Health Science Center at Houston, Houston, Texas, USA; b Institute of Molecular Medicine Center for Metabolic and Degenerative Diseases, McGovern Medical School at The University of Texas Health Science Center at Houston, Houston, Texas, USA; c Cell and Regulatory Biology Program of The University of Texas Graduate School of Biomedical Sciences, McGovern Medical School at The University of Texas Health Science Center at Houston, Houston, Texas, USA; d Molecular Signaling Section, Laboratory of Bioorganic Chemistry, National Institute of Diabetes and Digestive and Kidney Diseases, Bethesda, Maryland, USA

**Keywords:** CREB, DREADD, GPCR, bioluminescence imaging, cyclic AMP, glucose metabolism, liver

## Abstract

Hundreds of hormones and ligands stimulate cyclic AMP (cAMP) signaling in different tissues through the activation of G-protein-coupled receptors (GPCRs). Although the functions and individual effectors of cAMP signaling are well characterized in many tissues, pleiotropic effects of GPCR agonists limit investigations of physiological functions of cAMP signaling in individual cell types at different developmental stages *in vivo*. To facilitate studies of cAMP signaling in specific cell populations *in vivo*, we harnessed the power of DREADD (designer receptors exclusively activated by designer drugs) technology by creating *ROSA26*-based knock-in mice for the conditional expression of a Gs-coupled DREADD (rM3Ds-green fluorescent protein [GFP], or “GsD”). After Cre recombinase expression, GsD is activated temporally by the administration of the ligand clozapine *N*-oxide (CNO). In the same allele, we engineered a CREB-luciferase reporter transgene for noninvasive bioluminescence monitoring of CREB activity. After viral delivery of Cre recombinase to hepatocytes *in vivo*, GsD is expressed and allows CNO-dependent cAMP signaling and glycogen breakdown. The long-term expression of GsD in the liver results in constitutive CREB activity and hyperglycemia. *ROSA26*-Gs-DREADD mice can be used to study the physiological effects of cAMP signaling, acute or chronic, in liver or any tissue or cell type for which transgenic or viral Cre drivers are available.

## INTRODUCTION

G-protein-coupled receptors (GPCRs) represent the largest receptor family in mammals and regulate a wide range of physiological processes (1). Dysregulation of GPCR signaling underlies many diseases, including diabetes, cancer, hypothyroidism, hypogonadism, and fertility disorders (2–4). Due to their physiological actions, cell surface presentation, and diversity, GPCRs are among the largest classes of targets for drug development (5, 6). However, basic research on GPCR signaling as well as the identification of novel “druggable” pathways require effective tools that allow the specific manipulation of GPCR effector signaling in animals (7). Although pharmacological agonists or antagonists are widely used to regulate GPCR activity *in vivo*, it is challenging to attribute the resultant phenotypes to GPCR action in a specific cell type without the inclusion of tissue-specific receptor knockout mice. For example, β-adrenergic agonists stimulate cyclic AMP (cAMP) signaling in numerous tissues (8, 9) and regulate glucose production, lipolysis, thermogenesis, heart rate, and smooth muscle contraction, among other processes. Moreover, the activation of a single GPCR can sometimes stimulate more than one G protein (10–12). Such cross talk between different GPCR-activated pathways makes it impossible to selectively activate the major G protein classes using classical pharmacological tools.

To overcome these limitations, DREADDs (designer receptors exclusively activated by designer drugs) or RASSLs (receptors activated solely by synthetic ligands) (13–15) have been engineered to be insensitive to the endogenous ligand (acetylcholine) but highly responsive to the synthetic drug clozapine *N*-oxide (CNO) (16–19). DREADDs that selectively stimulate major families of G proteins, Gs, Gq, and Gi, have been created by making mutations that confer CNO sensitivity in Gi-coupled M4 or Gq-coupled M3 human muscarinic receptors or by replacing intracellular portions of the M3-muscarinic receptor with domains from the β1-adrenergic receptor that confer selectivity to Gs relative to Gq (20, 21). These DREADDs also activate β-arrestin signaling (22, 23). DREADDs have been widely used in animal models to study specific effects of GPCR signaling on numerous physiologic processes, including functions of different classes of neurons (24–26) as well as peripheral glucose metabolism (21, 24, 27, 28). Current approaches used to express DREADDs in specific tissues include the use of viruses and transgenic mice generated by random insertion or site-specific targeting (29). Viral delivery of DREADDs has the advantage of allowing rapid expression in many cell types. However, some cell types, such as muscle satellite cells, are refractory to viral infection *in vivo* (30–32), and it can be technically challenging to mark an entire cell lineage during development by using viral vectors. Random insertion transgenesis in mice to express DREADDs from tissue-specific promoters has offered an additional genetic approach but has different limitations, including unknown copy numbers, random genomic insertion sites, and a lack of flexibility in using the same animal line for expression in different tissues. To circumvent these challenges and offer complementary approaches, other groups have created *ROSA26*-based knock-in mice with a Cre-releasable lox-stop-lox (LSL) cassette to express Gi- or Gq-coupled DREADDs in different cell types by using Cre recombinase (33, 34). However, no such knock-in mouse line for the Cre-inducible expression of a Gs-selective DREADD is currently available.

Here we describe the first *ROSA26* knock-in mouse for the tissue-specific expression of a Gs-coupled DREADD (rM3Ds-DREADD-green fluorescent protein [GFP], referred to here as “GsD”). This mouse line allows the Cre-dependent expression of Gs-DREADD using tissue-specific Cre drivers or Cre-expressing viruses. The version of Gs-DREADD that we employed has relatively low basal activity *in vitro* and does not activate Gq signaling (21, 24). Additionally, we engineered a CREB-activated luciferase reporter within the same allele; this reporter is not under the control of Cre recombinase and allows the monitoring of CREB activity by noninvasive bioluminescence imaging *in vivo* or luciferase reporter assays *in vitro* (35–37). One of the best-understood physiological effects of cAMP signaling is the promotion of hepatic glucose output through glycogen breakdown and gluconeogenesis (38, 39). Therefore, we expressed Cre recombinase in liver and primary mouse hepatocytes to characterize the *ROSA26*-GsD mouse line.

## RESULTS

### Generation of *ROSA26-LSL-GsDREADD-CRE-luc* knock-in mice.

**FIG 1:**
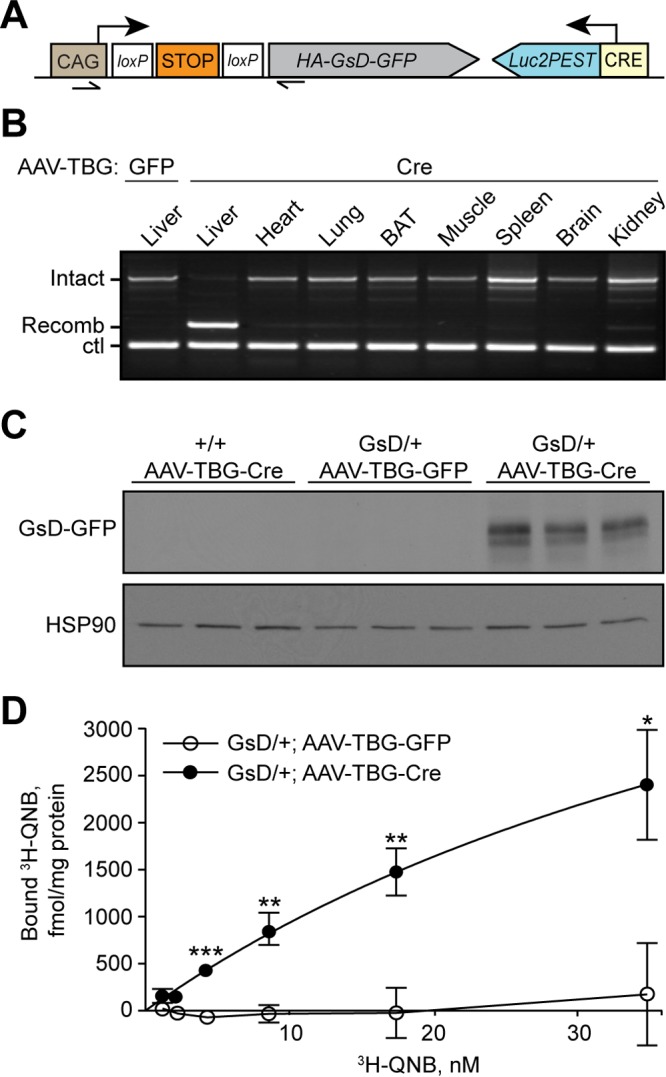
Gs-DREADD-Luc mice express Gs-DREADD (GsD) in liver after Cre recombinase expression. (A) Schematic of the *ROSA26-LSL-GsDREADD-CRE-luc* allele. Arrowheads, PCR primers; arrows, transcriptional start sites. (B) Genomic PCR showing liver-specific recombination of the *ROSA26-GsD* locus after AAV-TBG-Cre infection *in vivo* compared with AAV-TBG-GFP infection (lane 1). PCR products correspond to intact and recombined LSL cassettes amplified by the primers indicated in panel A. Ctl, *Creb1* genomic DNA (control). (C) Western blot analysis of GsD (anti-GFP) and HSP90 in livers of mice infected with AAV-TBG-GFP or AAV-TBG-Cre (*n* = 3 per group). (D) [^3^H]QNB binding assay on membranes isolated from livers of GsD mice infected with AAV-TBG-GFP or AAV-TBG-Cre (means ± standard errors of the means; *n* = 3 per group) (*, *P* < 0.05; **, *P* < 0.01; ***, *P* < 0.001 [by a *t* test]).

To stimulate cAMP production *in vivo*, we generated *ROSA26* knock-in mice for Cre-inducible expression of a Gs-DREADD, hemagglutinin (HA)-rM3Ds-enhanced GFP (eGFP) (GsD). N-terminal HA and C-terminal GFP tags were added to facilitate the detection of DREADD (24). The C-terminal GFP tag reduced basal DREADD activity observed in a prior GsD (21, 24). To create a mouse line for the facile expression of GsD in any cell type, we used the same strategy as that used for the *ROSA26-LSL* Cre reporters in which fluorescent proteins are expressed from a CAG promoter after the Cre-mediated excision of an LSL cassette (40). We replaced the *tdTomato* cDNA within the Ai9 *ROSA26* targeting vector (40) with GsD. This allele uses the CAG promoter, a well-characterized synthetic promoter giving high levels of expression in mammalian cells (41), and a lox-stop-lox (LSL) cassette with transcriptional splice acceptors and translational stop sites to repress the expression of the downstream gene until Cre recombinase is expressed. To monitor GsD activity in the same animals, we incorporated a CREB-activated luciferase reporter construct (35) in the reverse orientation; the CREB reporter is not dependent on Cre recombinase expression (Fig. 1A).

cAMP signaling is well known to drive hepatic glucose production and CREB activity (42–44). In addition, viral and transgenic CREB reporters are highly sensitive to fasting in liver (35, 36), and hepatocytes are efficiently infected with adenovirus (AdV) and adeno-associated virus (AAV) vectors (45, 46). We therefore injected mice with AAVs encoding Cre recombinase or GFP under the transcriptional control of the thyroxin binding globulin (TBG) promoter (AAV-TBG-GFP or -Cre) to express GsD in hepatocytes. Three weeks after AAV injection, the stop cassette was efficiently and specifically removed from genomic DNA of livers of AAV-Cre-infected mice (Fig. 1B). We readily detected the GFP-tagged receptor in liver homogenates of AAV-TBG-Cre-infected mice by Western blotting (Fig. 1C). Native GFP fluorescence was not visible by fluorescence microscopy in hepatocytes prepared from these animals, similarly to the AAV-mediated expression of the same GsD-GFP construct in AgRP (agouti-related protein) neurons (24). To determine hepatic GsD expression levels, we performed radioligand binding assays using ^3^H-labeled quinuclidinyl benzilate (QNB), a muscarinic receptor antagonist. We detected strong [^3^H]QNB binding to the membrane fraction from livers of mice infected with AAV-TBG-Cre (GsD density at the highest [^3^H]QNB concentration used of 2.40 ± 0.59 pmol/mg membrane protein; *n* = 3) but not with GFP control membranes (Fig. 1D). DREADD expression levels were similar to those achieved in brain by using a Tet-on GqD transgenic mouse line (25).

### Gs-DREADD stimulates CREB in hepatocytes.

**FIG 2:**
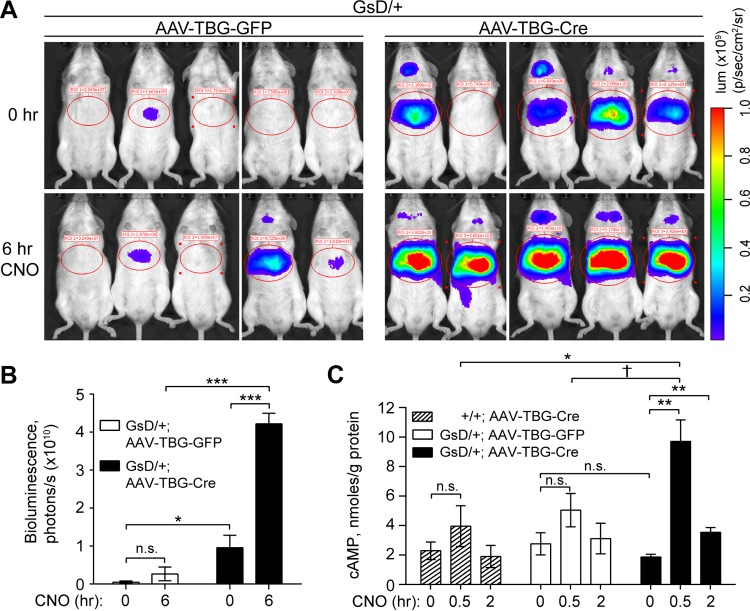
GsD activation stimulates CREB activity in liver *in vivo*. (A) Bioluminescence (lum) imaging of GsD mice at baseline (0 h) and repeated on the same animals after CNO injection (1 mg/kg) (6 h). p, surface radiance in photons. (B) Quantification of bioluminescence in the liver regions circled in panel A (means ± standard errors of the means; *n* = 5 per group). (C) cAMP levels in livers of mice of the indicated genotypes 5 to 8 weeks after AAV infection and control or CNO injection (means ± standard errors of the means; *n* = 3 to 5 per group). (*, *P* < 0.05; **, *P* < 0.01; ***, *P* < 0.001; †, *P* = 0.07).

DREADDs are activated by an otherwise pharmacologically inert ligand, CNO. We first tested *in vivo* GsD activity by measuring the bioluminescence of the genomic CREB-activated luciferase reporter. Under basal conditions, control mice showed little CREB-activated bioluminescence, but GsD animals infected with AAV-TBG-Cre had elevated CREB activity without CNO injection (Fig. 2A and B). Six hours after CNO injection, hepatic bioluminescence increased 4-fold in Cre-infected, but not in GFP-infected, GsD-expressing livers (Fig. 2A and B). Basal cAMP levels were not elevated in GsD-expressing livers but increased acutely (30 min) and gradually declined within 2 h of CNO injection, demonstrating a transient effect of GsD activation on hepatic cAMP production (Fig. 2C). Levels of basal CREB-activated luciferase activity and the CREB target genes *Sik1* (47) and *Pgc-1*α (42) were elevated in GsD-expressing livers (Fig. 2A and B; see also Fig. S1 in the supplemental material), but this was not observed at the level of endogenous cAMP. This discrepancy is likely due to the different degradation kinetics of cAMP, mRNA, and luciferase protein.

**FIG 3:**
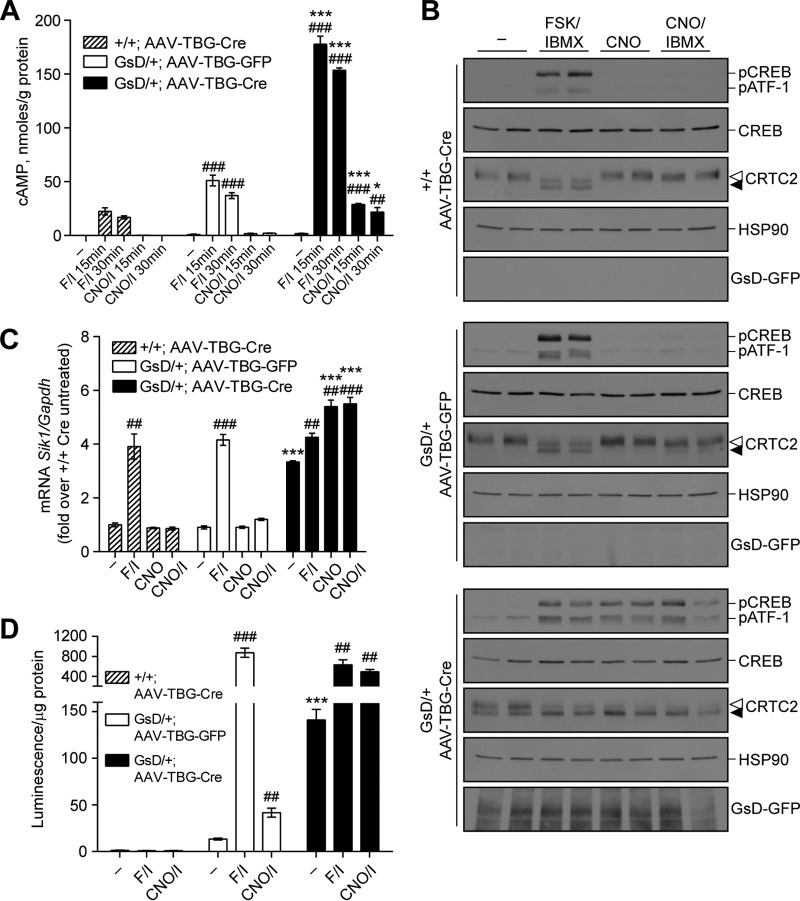
GsD activation stimulates the cAMP-CREB pathway in primary mouse hepatocytes. Primary hepatocytes were prepared from +/+ or GsD/+ mice that were injected with AAV-TBG-GFP or AAV-TBG-Cre 3 weeks earlier. Hepatocytes were treated with FSK (10 μM)-IBMX (18 μM), CNO (10 μM), or CNO (10 μM)-IBMX (18 μM). (A) cAMP levels after 15 or 30 min of the indicated treatments (means ± standard errors of the means; *n* = 3). (B) Western blots of phospho-CREB (S133), total CREB, CRTC2, HSP90, and GsD-GFP in hepatocytes treated for 15 min as indicated. Open arrowhead, phospho-CRTC2 (inactive); filled arrowhead, dephospho-CRTC2 (active). Duplicates are shown (quantified in Fig. S2A in the supplemental material). (C) *Sik1* mRNA levels in hepatocytes treated as indicated for 1 h (means ± standard errors of the means; *n* = 3). (D) Luciferase activity in hepatocytes treated as indicated for 4 h (*n* = 3). #, significance versus untreated controls of the same genotype; *, significance for GFP-Cre within each treatment group. Data are representative of results from 2 or 3 independent experiments performed on cells harvested from separate animals. *, *P* < 0.05; **, *P* < 0.01; ***, *P* < 0.001 (by *t* tests).

To demonstrate that the GsD-dependent signaling that we observed in liver tissue is autonomous to hepatocytes and to investigate the signaling properties in more detail, we isolated primary hepatocytes from GsD mice previously infected with AAV-TBG-GFP or AAV-TBG-Cre. In Cre-expressing GsD hepatocytes, CNO, CNO-isobutylmethylxanthine (IBMX), and forskolin (FSK)-IBMX (adenylyl cyclase agonist and phosphodiesterase inhibitor) each stimulated cAMP production (Fig. 3A), CREB(S133) phosphorylation, and dephosphorylation of CREB-regulated transcription coactivator 2 (CRTC2) (Fig. 3B). As expected, control GFP-expressing GsD hepatocytes were insensitive to CNO but sensitive to FSK-IBMX (Fig. 3). cAMP production in response to CNO-IBMX was maximal by 15 min and sustained through 30 min. The adenylyl cyclase agonist FSK induced more cAMP production in GsD-expressing hepatocytes than in control hepatocytes that did not express GsD (Fig. 3A). This is expected, as Gs-alpha and FSK synergistically stimulate adenylyl cyclase (48–50), and we expect that at least a small amount of Gs-alpha is activated by GsD overexpression. Interestingly, FSK-IBMX, CNO, and CNO-IBMX stimulated similar levels of CREB phosphorylation on Ser133 in GsD-expressing hepatocytes (Fig. 3B; see also Fig. S2A in the supplemental material), indicating that CREB phosphorylation was maximal at this dose of FSK-IBMX. However, the CREB coactivator CRTC2, which is dephosphorylated and translocates to the nucleus in response to cAMP signaling (47), was already partially dephosphorylated (activated) in GsD-expressing hepatocytes and further dephosphorylated upon FSK-IBMX or CNO-IBMX treatment (Fig. 3B; Fig. S2A). CRTC2 activation may therefore partly explain why GsD-expressing hepatocytes have stronger transcriptional responses to FSK-IBMX than do control hepatocytes with equivalent levels of phospho-CREB (pCREB). Consistent with cAMP levels, CREB target genes were induced by FSK-IBMX irrespective of the genotype (Fig. 3C; Fig. S2B to D), and GsD expression increased basal mRNA expression levels of these genes (*Sik1*, *G6pase*, *Pgc-1*α, and *Pepck*). Besides *G6pase*, the transcription of CREB target genes was further induced by CNO-IBMX in GsD-expressing hepatocytes (Fig. 3C; Fig. S2B to D). In keeping with these observations, CREB reporter activity was elevated by GsD expression and further induced by CNO-IBMX (Fig. 3D). Together, these data show that GsD expressed from the *ROSA26*-based allele in hepatocytes exhibits basal signaling sufficient to stimulate the cAMP-CREB pathway; this pathway is further enhanced by the ligand CNO.

We confirmed these results in primary GsD-expressing hepatocytes acutely infected *ex vivo* with AdV encoding GFP or Cre. Twenty-four hours after infection, cAMP levels were elevated in GsD-expressing cells and further enhanced by CNO-IBMX by 15 min (see Fig. S3A in the supplemental material). CNO-IBMX had no effect on hepatocytes infected with AdV-GFP. Similarly to AAV-infected hepatocytes, FSK-IBMX elicited at least a 5-fold-higher cAMP response than did CNO-IBMX in AdV-Cre-infected hepatocytes. We also observed CNO-dependent CREB phosphorylation and target gene induction in GsD-expressing cells after AdV-Cre infection but not after AdV-GFP infection (Fig. S3B to E). Similar to the case for AAV-infected hepatocytes, we found that FSK-IBMX had a stronger effect on CREB target genes in primary hepatocytes expressing GsD than in control hepatocytes (Fig. S3D and E), again demonstrating cooperativity between Gs-alpha and FSK.

### Hepatic GsD expression elevates blood glucose levels.

In liver, the fasting hormones glucagon and epinephrine stimulate Gs-coupled GPCRs to activate cAMP signaling (51). These hormones comprise a major portion of the counterregulatory response to hypoglycemia and contribute to the maintenance of blood glucose in the fasted state. Cyclic AMP-dependent protein kinase (PKA) drives glycogen breakdown by activating glycogen phosphorylase and inhibiting glycogen synthase (52). PKA also induces gluconeogenesis by the activation of CREB and the subsequent induction of genes encoding rate-limiting gluconeogenic enzymes and transcriptional coactivators (42, 53–55). Although CREB-dependent expression of gluconeogenic genes is essential to support glucose production during fasting (42), it is detrimental in type 2 diabetes, when excessive CREB/CRTC2 complex activity contributes to hyperglycemia (36, 56).

**FIG 4:**
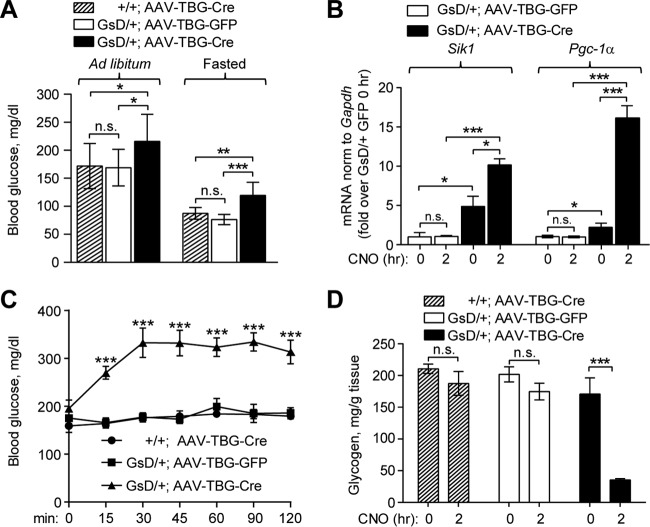
Expression of Gs-DREADD in liver elevates blood glucose levels. (A) Blood glucose levels in *ad libitum*-fed and fasted mice (means ± standard deviations; *n* = 10 per group). (B) *Sik1* and *Pgc-1*α mRNA levels in mouse livers 5 to 8 weeks after AAV injection, normalized to *Gapdh* levels, expressed relative to values for AAV-TBG-GFP at 0 h. (C) Blood glucose levels after CNO injection (1 mg/kg s.c.) in *ad libitum*-fed mice (means ± standard errors of the means; *n* = 5 per group). (D) Liver glycogen levels in *ad libitum*-fed mice 0 h and 2 h after CNO injection (1 mg/kg s.c.) (means ± standard errors of the means; *n* = 3 to 5 mice per group). *, *P* < 0.05; **, *P* < 0.01; ***, *P* < 0.001; n.s., not significant (by *t* tests or ANOVA).

We therefore tested the impact of hepatic GsD activation on blood glucose levels. GsD mice expressing Cre had increased blood glucose levels compared to those in control animals in both the *ad libitum*-fed and fasted states, even without CNO delivery (Fig. 4A), in keeping with the increase in basal CREB activation that we observed in GsD-expressing mice (Fig. 2A and B). Increased fasting glucose levels were mirrored by elevated expression levels of hepatic *Pgc-1*α mRNA (Fig. 4B), indicating that cAMP-dependent transcription of progluconeogenic genes likely contributes to hyperglycemia in mice with GsD expression in liver. Similarly to *Pgc-1*α, the CREB target gene *Sik1* is also constitutively expressed in GsD-expressing livers, and both genes are further induced acutely by CNO injection (Fig. 4B). Activation of the cAMP-PKA pathway in liver by glucagon results in rapid glycogen breakdown and the release of glucose into the bloodstream (44, 52, 57). Similarly to glucagon (57), CNO rapidly increased blood glucose levels, with near doubling within 30 min of injection (Fig. 4C). As expected, CNO did not affect blood glucose in control mice (Fig. 4C). This acute effect was mostly due to CNO-stimulated glycogen breakdown, as the hepatic glycogen level was dramatically reduced 2 h after GsD activation with CNO (Fig. 4D).

## DISCUSSION

DREADD technology enables the selective initiation of Gq-, Gi-, and Gs-mediated signaling to study cellular and physiological consequences of activating these pathways. Previous work used DREADDs to elucidate the effects of G-protein-mediated signaling in the regulation of neuronal activity, glucose metabolism, and mitogenic signaling, to name a few (21, 24, 27, 28, 58). We created GsD-CRE-Luc knock-in animals to achieve chemical-genetic control of the cAMP signaling pathway with the opportunity for a noninvasive readout of cAMP pathway activity by using bioluminescence imaging. Because this mouse line allows Cre-dependent GsD expression, it will be useful for the study of cAMP signaling in any cell type into which Cre can be delivered by transgenic expression, virus delivery, or direct DNA transduction. This enables chemical-genetic control of cAMP signaling not only in specific cell types but also at specific developmental stages using inducible Cre lines or acute virus delivery, thereby avoiding developmental effects of Gs signaling.

To characterize this mouse line, we used viral vectors expressing Cre to induce the expression of GsD and tested the effects of GsD activation on well-characterized cAMP effector pathways in liver. As expected, the expression of Cre allowed GsD expression in hepatocytes, and the DREADD agonist CNO triggered cAMP-PKA signaling, glycogen breakdown, CREB activation, and CREB target gene expression in livers of GsD mice. These molecular alterations were associated with increased blood glucose levels in GsD mice in both fed and fasted states even without CNO injection, indicating that the expression level of GsD accomplished in these mice most likely increased the cAMP signaling tone in liver and, hence, elevated hepatic glucose production. Nonetheless, CNO injection further stimulated acute glucose excursion and hepatic glycogen depletion, mimicking the effects of glucagon on liver (44, 47).

Radioligand binding studies demonstrated that hepatic GsD expression levels were relatively high (Fig. 1D). It is well known that GPCRs that are expressed at high levels can display significant ligand-independent activity (59). Thus, it is likely that mice that express GsD at lower levels will show no or greatly reduced basal DREADD activity. Consistent with this concept, no constitutive DREADD signaling was observed after the expression of GsD in AgRP neurons of the hypothalamus (24). In the present experiments, we show that GsD expressed in hepatocytes using AAV or adenoviral Cre exhibits basal activity sufficient to stimulate the CREB pathway. In this system, basal GsD activity is sufficient to moderately stimulate hepatic glucose production, but the ligand CNO still has a strong effect on promoting acute cAMP-dependent responses, including glycogen breakdown and CREB target gene expression. Future studies employing this animal line to express GsD in different tissues should therefore incorporate control animals to assess basal GsD activity, and data should be interpreted carefully.

Hyperglucagonemia is thought to contribute to hyperglycemia in type 2 diabetes (60–63). The GsD model will allow the simulation of glucagon-like signaling specifically in hepatocytes independently of other metabolic changes associated with type 2 diabetes, such as hepatic lipid accumulation or pleiotropic effects of glucagon receptor signaling on metabolism (64), including direct actions on extrahepatic tissues such as brain (65). Some of the salutary effects of the antidiabetic drug metformin have been linked to reduced hepatocyte cAMP signaling through several mechanisms (66–68). The GsD line could be an excellent tool to further study the complex regulation of cAMP signaling pathways in hepatic metabolism.

## MATERIALS AND METHODS

### Generation of *ROSA26-LSL-GsDREADD-CRE-luc* mice.

A fragment encoding HA-rM3Ds-eGFP (Gs-coupled DREADD) was excised from the pcDNA5-Rs-eGFP plasmid (generated by J. Wess) using BamHI/NotI, cloned into a custom pUC57-based FseI shuttle vector, and subcloned into Ai9 (*ROSA26-lsl-tdTomato* targeting vector) (40) using FseI sites. The CREB-activated luciferase reporter [CRE-Luc2P-simian virus 40 (SV40) poly(A)] was subcloned from pGL4.29 (Promega) (GenBank accession number DQ904461.1) using BamHI/SpeI into a custom pBluescript vector containing two XmaI sites (pBS-Xma2) and subsequently subcloned into the Ai9/rM3 vector using XmaI sites (35). This insert contains four full CREB binding sites, one half-site, a minimal promoter containing a TATA box, *Luc2P* cDNA (codon-optimized firefly luciferase [*Luc2*] fused to a PEST domain (enriched in Pro, Glu, Ser, and Thr) to enhance turnover), and a polyadenylation signal. The resulting targeting construct, pAi9-GsD-CRE-luc, was confirmed by sequencing, linearized with SgrDI, purified, and electroporated into mouse 129/SvImJ embryonic stem (ES) cells. Clones were screened by Southern blotting with probes to the 5′ (865 bp; amplified by ROSA-4F [5′-GTAGGCAATACCCAGGCAAA] and ROSA-4R [5′-GAGTCCCGATCCCCTACCTA]) and 3′ (779 bp; amplified by ROSA-2F [5′-TGGCACTGTTCATTTGTGGT] and ROSA-2R [5′-TTTGGATGGTTTTTGCATCA]) genomic regions after DNA digestion with EcoRI or EcoRV, respectively. Mouse ES work, clone regrowth, and clone injection were performed by the Transgenic and Stem Cells Service Unit at The University of Texas Health Science Center at Houston. Chimeric (founder) male mice were bred with albino female *C57BL/6J-Tyr*^*c-2J*^*/J* (“albino BL6”) mice (Jackson). Agouti pups containing the transgene were used as founders. Animals were back-crossed to albino BL6 mice for at least 3 generations; albino transgenic animals were chosen for further breeding. The official strain nomenclature is Gt(ROSA)26Sor⟨tm1(CAG-Chrm3*/GFP,cAMPRE-luc)Berd⟩, with the allele synonym *ROSA26*-GsD-Luc and Mouse Genome Informatics (MGI) accession number 5696731 (referred to here as GsD). Male heterozygous or homozygous GsD animals were bred to wild-type females to generate cohorts. Genotyping primers were used for the *ROSA26*^*GsD*^ allele (206-bp product, amplified by ROSA-9F [5′-CTCGAAGTACTCGGCGTAGG] and ROSA-9R [5′-CTCGAAGTACTCGGCGTAGG]]) and the *ROSA26*^+^ wild-type (WT) allele (297-bp product, amplified by oIMR9020 [5′-AAGGGAGCTGCAGTGGAGTA] and oIMR9021 [5′-CCGAAAATCTGTGGGAAGTC]) (40).

### Mouse experiments.

All animal procedures were approved by the Animal Welfare Committee of The University of Texas Health Science Center at Houston (approval AWC-14-0071). Mice were housed at 22°C in individually ventilated cages with free access to food and water with a 12-h light/dark cycle (7 a.m./7 p.m.). Male heterozygous *ROSA26*^GsD/+^ mice were used for most studies; female mice were used for the data shown in Fig. S1 in the supplemental material. Albino mice were used for bioluminescence imaging. Mice aged 8 to 10 weeks were injected intravenously (i.v.) with 2 × 10^11^ genomes of recombinant AAV2/8-TBG-Cre (AAV8.TBG.PI.Cre.rBG) or AAV2/8-TBG-GFP (AAV8.TBG.PI.eGFP.WPRE.bGH) (Penn Vector Core, University of Pennsylvania). Three weeks later, experiments commenced. For CNO tolerance tests, CNO (1 mg/kg of body weight in 0.9% sterile saline) was injected subcutaneously (s.c.) into *ad libitum*-fed mice. The level of blood glucose from tail bleed samples (fasted for 16 h from 5 p.m. to 9 a.m. or *ad libitum* fed between 7 a.m. and 12 p.m.) was measured by using a glucometer (One Touch Ultra). Animals were euthanized by CO_2_ or by exsanguination under isoflurane anesthesia. Liver tissue was immediately excised, flash frozen in liquid N_2_, and stored at −80°C. Where indicated, primary hepatocytes were harvested from AAV-infected animals 3 weeks after AAV injection.

### Bioluminescence reporter imaging.

Bioluminescence imaging was performed on mice prior to and 6 h after CNO injection (1 mg/kg s.c.) as described previously (35, 36). Mice were anesthetized by isoflurane inhalation, injected intraperitoneally (i.p.) with d-luciferin (100 mg/kg), and transferred to the heated stage (37°C) of an Ivis Lumina XR instrument (Caliper Life Sciences) for imaging (F-stop 1, binning 4). Image analysis was performed by selecting regions of interest in the liver to quantify luminescence signals by using Caliper LivingImage software. Pseudocolored luminescence images were overlaid onto photographs of the same animals and exported by using matched visualization settings. Total bioluminescence flux was plotted on a linear scale.

### Analysis of liver tissue.

Frozen liver was pulverized under liquid N_2_ by using a mortar and pestle and homogenized by using a rotor stator in the appropriate lysis buffer for analysis of genomic DNA (PCR), RNA (quantitative PCR [qPCR]), protein (Western blotting), and glycogen (biochemical assay) as described previously (69). For [^3^H]QNB (a high-affinity muscarinic antagonist) binding assays, membranes were prepared from frozen liver and incubated with [^3^H]QNB as described previously (70). The mRNA level was normalized to the *Gapdh* level, expressed as fold changes over the control.

### Primary hepatocytes.

Hepatocytes were isolated from anesthetized mice by hepatic perfusion with collagenase as described previously (69, 71). Where indicated, cells were infected with adenovirus (AdV-GFP or AdV-Cre) for 24 h. Cells were stimulated with FSK (10 μM)-IBMX (18 μM), CNO (10 μM), or CNO (10 μM)-IBMX (18 μM).

### Antibodies.

The following antibodies were used for Western blotting: pCREB (87G3) (catalog number 9198; Cell Signaling), CREB (48H2) (catalog number 9197; Cell Signaling), CRTC2 [EPR3384(2)] (catalog number ab109081; Abcam), GFP (catalog number 600-101-215; Rockland), and HSP90 (catalog number sc-7947; Santa Cruz).

### cAMP assay.

Primary hepatocytes and livers were frozen in liquid N_2_. Twenty micrograms of total protein was assayed by using the Direct cAMP enzyme-linked immunosorbent assay (ELISA) kit (Enzo Life Sciences) according to the manufacturer's instructions. The cAMP level was normalized to the protein concentration.

### Statistical analysis.

Densitometry on Western blots was analyzed by using ImageJ. pCREB and total CREB were run on separate gels in parallel. pCREB and total CREB values were normalized to those for the HSP90 loading controls on the same gel, and the normalized values were used to determine the pCREB/CREB ratio. Normalized fold changes were tested between treatments by using a *t* test. Other data were analyzed by using two-tailed Student's *t* test for comparisons of averages of groups or two-way repeated-measures analysis of variance (ANOVA) with Tukey's posttest with multiple comparisons (CNO tolerance test) by using GraphPad Prism.

## Supplementary Material

Supplemental material
