# Supplementary material for: Gs-DREADD Knock-In Mice for Tissue-Specific, Temporal Stimulation of Cyclic AMP Signaling
Source: Mol Cell Biol. 2017 Apr 14;37(9):e00584-16. doi: 10.1128/MCB.00584-16 (PMC5394278; doi:10.1128/MCB.00584-16)
Supplement: Supplemental material [file supp_37_9_e00584-16__index.html]

Supplemental material 

# Gs-DREADD Knock-In Mice for Tissue-Specific, Temporal Stimulation of Cyclic AMP Signaling

## Supplemental material

- Supplemental file 1 -

  Fig. S1 (Basal CREB target gene expression in livers of GsD; AAV-TBG-Cre mice), S2 (CNO response of primary hepatocytes from GsD; AAV-TBG-Cre mice), and S3 (GsD activation and cAMP-CREB pathway in primary mouse hepatocytes)

  PDF, 2.0M
