# Supplementary material for: Gs-DREADD Knock-In Mice for Tissue-Specific, Temporal Stimulation of Cyclic AMP Signaling
Source: Mol Cell Biol. 2017 Apr 14;37(9):e00584-16. doi: 10.1128/MCB.00584-16 (PMC5394278; doi:10.1128/MCB.00584-16)
Supplement: Supplemental material [file MCB.00584-16_zmb009171468s1.pdf]

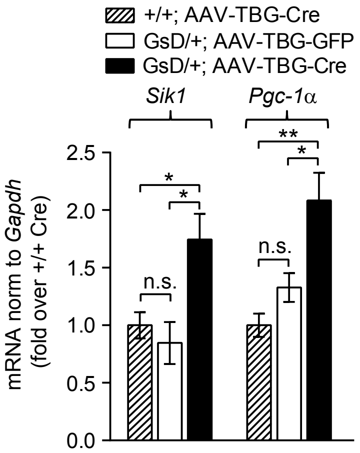

Supplementary Figure 1. Basal CREB target gene expression is elevated in livers of GsD; AAV-TBG-Cre mice. *Sik1* and *Pgc-1*α mRNA normalized to *Gapdh*, expressed relative to +/+; AAV-TBG-Cre mice; mean ±SEM, n=4-5 per group, *p<0.05, **p<0.01, ***p<0.001 by t-tests.

Supplemental material
Akhmedov, et al.
Gs-DREADD knock-in mice for tissue-specific, temporal stimulation of cAMP signaling

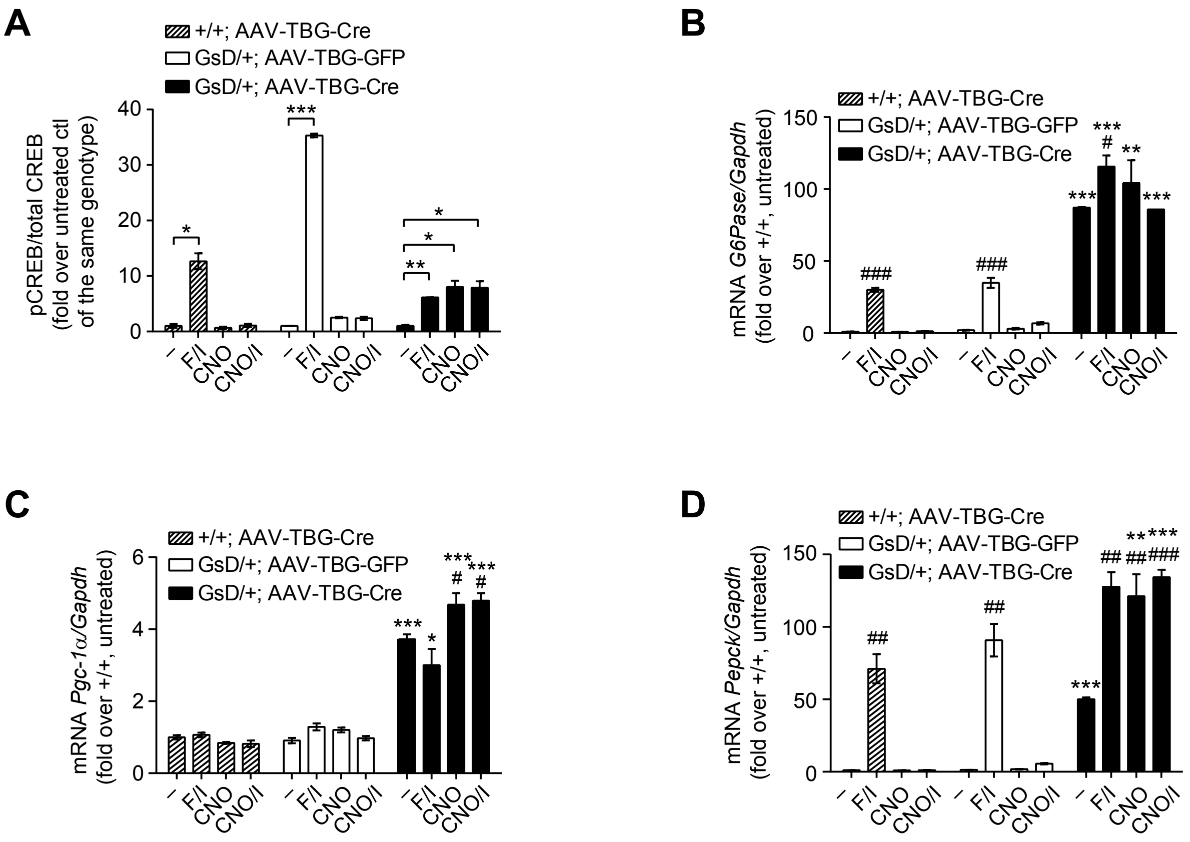

Supplementary Figure 2. Primary hepatocytes from GsD; AAV-TBG-Cre mice, but not control mice, respond to CNO. A) Quantification of pCREB western blots shown in Fig 3B. B, C, D) Expression of CREB target genes *G6pase* (B), *Pgc-1α* (C) and *Pepck* (D) in the same samples shown in Fig 3C; mean ± SEM, #: significance vs untreated ctl within the same genotype *: significance GFP: Cre within each treatment group,*p<0.05, **p<0.01, ***p<0.001 by t-tests.

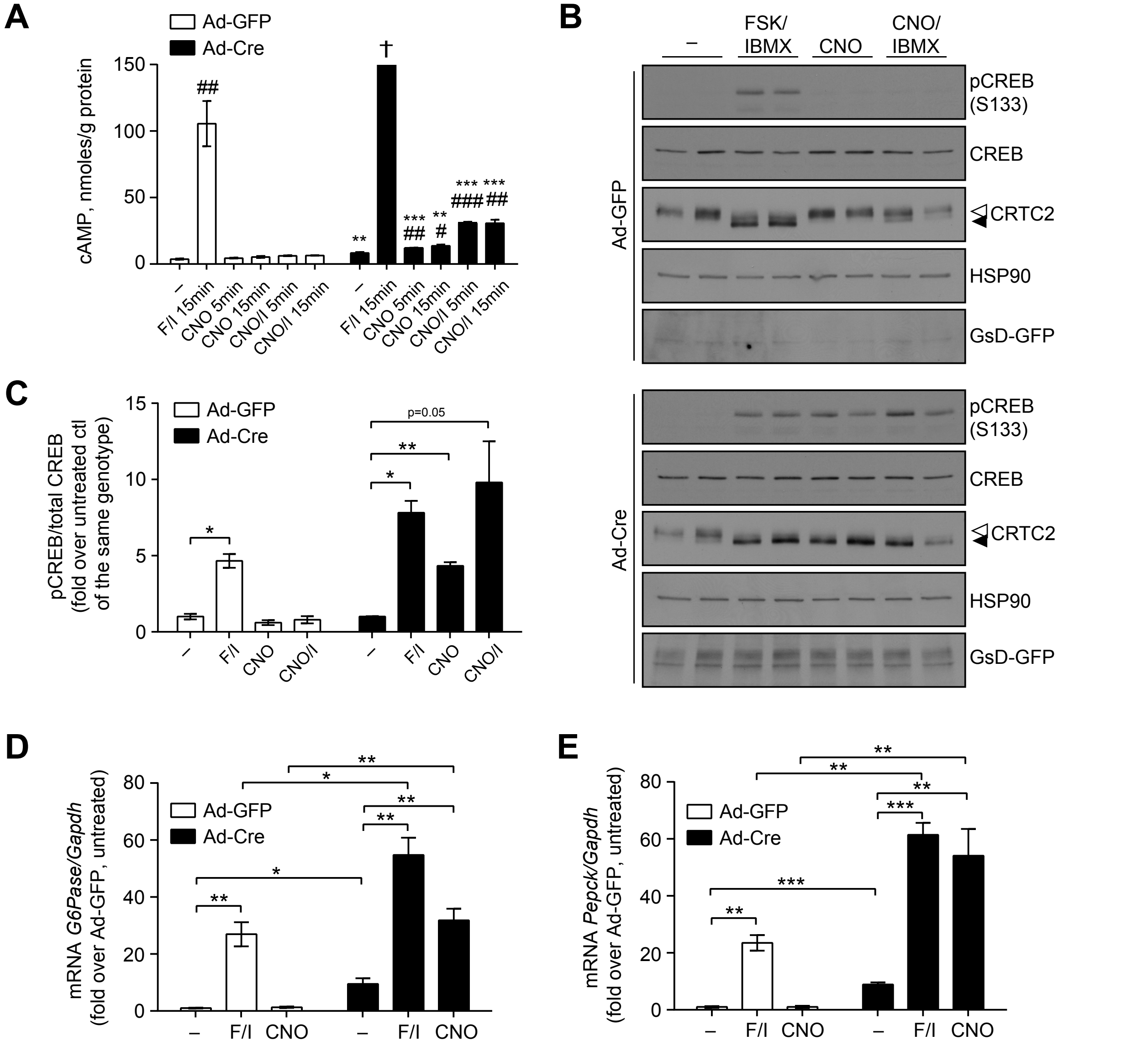

Supplementary Figure 3. GsD activation stimulates the cAMP-CREB pathway in primary mouse hepatocytes. Primary hepatocytes were prepared from GsD/+ mice, infected with Ad-GFP or Ad-Cre *ex vivo* and treated with CNO (10 µM), CNO (10 µM) /IBMX (18 µM) or FSK (10 µM)/ IBMX (18 µM). A) cAMP after 5 or 15 min of indicated treatments; mean ±SEM; n=3. †: cAMP levels were above the measurement range. #: significance vs untreated ctl within the same genotype *: significance GFP: Cre within each treatment group. B) Western blots of phospho-CREB(S133), total CREB and CRTC2 in hepatocytes treated for 15 min as indicated. Open arrowhead, phospho-CRTC2 (inactive); filled arrowhead, dephospho-CRTC2 (active). Duplicates shown; panel is representative of 3 independent experiments performed in duplicate. C) Quantification of pCREB western blots shown in (B). D) *G6Pase* and E) *Pepck* mRNA in hepatocytes treated with FSK (10 µM)/ IBMX (18 µM) or CNO (10 µM) (1 hr); mean ± SEM. *p<0.05, **p<0.01, ***p<0.001 by t-tests; representative of 3 independent experiments performed in triplicate.
